# Genome Elimination: Translating Basic Research into a Future Tool for Plant Breeding

**DOI:** 10.1371/journal.pbio.1001876

**Published:** 2014-06-10

**Authors:** Luca Comai

**Affiliations:** Plant Biology and Genome Center, University of California Davis, Davis, California, United States of America; The University of North Carolina at Chapel Hill, United States of America

## Abstract

During the course of our history, humankind has been through different periods of agricultural improvement aimed at enhancing our food supply and the performance of food crops. In recent years, it has become apparent that future crop improvement efforts will require new approaches to address the local challenges of farmers while empowering discovery across industry and academia. New plant breeding approaches are needed to meet this challenge to help feed a growing world population. Here I discuss how a basic research discovery is being translated into a potential future tool for plant breeding, and share the story of researcher Simon Chan, who recognized the potential application of this new approach—genome elimination—for the breeding of staple food crops in Africa and South America.

## Abstract 2

Genome elimination exemplifies how a basic research discovery can be translated into a future tool for plant breeding. As Luca Comai explains in his Perspective, it was Simon Chan who recognized its potential for improving the breeding of staple food crops.

This article is part of the *PLOS Biology* Collection “The Promise of Plant Translational Research.”

## Introduction

Humanity exists in a precarious balance between bounty and famine, and the long-term sustainability of current human society is dependent on our ability to breed crop varieties adapted to our changing environment. We have made considerable progress towards this end, as reflected in the recent history of crop improvement. This history can be divided into three periods that are characterized by different technological approaches: 1940–1980, crop modification through the breeding of yield-enhancing traits (a strategy that underpinned the green revolution) [1]; 1980–2000, the use of transgenesis to introduce single-gene traits and to facilitate weed and pest control [2]–[4]; 2000–present, the use of whole genome sequence data [5]–[7], as well as a deeper understanding of genetic and epigenetic mechanisms [8], to facilitate new technologies and approaches to yield another leap forward in agricultural productivity.

These periods of crop improvement have taken place against a backdrop of different socio-economical climates. The green revolution addressed an impending Malthusian catastrophe in the developing world by rationalizing the means of agricultural productivity. This entailed combining optimized agronomic practices with crop varieties that being shorter and stiffer tolerated higher nitrogen without lodging (when the stalk of a plant bends). Success required the acceptance and sharing of this technological platform between scientists, local government, and farmers. The introduction of genetically modified (GM) crops, on the other hand, was largely enabled by the efforts of selected agricultural biotechnology companies, which saw the advantages that could be reaped by introducing into plants genes encoding herbicide and pest tolerance [4]. While the cultivation of GM crops has brought considerable benefits to some farmers in terms of efficient weed and pest control [2], their development comes with the large costs of regulatory compliance, combined with widespread public diffidence and frequent opposition [9]. This social context requires that each transgenic modification crosses a critical threshold of economic value. In addition, given the regulatory cost of implementing a new transgenic trait [10], farmers and plant breeders have limited ability to explore and to develop new transgenic resources to address localized problems concerning agriculture and cultural preferences for staple foods.

Over recent years, it has become clear that future crop improvement efforts will require approaches that are easy to access and that lend themselves to addressing local challenges, while empowering discovery across industry, academia, and the farming community. Plant breeding meets these criteria. Its successes are awe-inspiring: consider the difference between maize and its wild form, teosinte, or between polyploid bread wheat and its parental species. They are as remarkable as the difference between a chihuahua and its wild wolf ancestor (although perhaps in the opposite way). Notably, selective plant breeding can be practiced with success by a Neolithic analphabet human ancestor or by a PhD-toting scientist, and the former has clearly the edge in achievement. Given time, plant breeding can yield miracles. Time, however, we do not have in the face of our growing population, dwindling resources, and changing climate. We need to re-invent plant breeding now to make it more efficient, more powerful, and faster.

## Genome Elimination

An important contribution to this endeavor was made by Simon Chan and colleagues in 2010 [11], 2011 [12], and 2012 [13],[14]; notwithstanding the fact that his training in basic biology had little to do with plant breeding. Chan had studied telomerase function in yeast for his graduate work, DNA methylation in plants for his postdoctoral work, and, while starting a faculty career at the University of California at Davis, decided to explore centromere determination and function in the model plant, *Arabidopsis*. Centromeres are DNA regions on which kinetochores are formed. These are the handles to which, during mitotic and meiotic cell divisions, spindle fibers are attached to drag chromosomes through the mother cell and partition them to the daughters. While most regulatory DNA regions are determined by specific nucleotide sequences, centromeres depend on an epigenetic signal, that is, a persistent DNA modification that does not depend on sequence. This largely mysterious epigenetic signal requires a variant histone H3, called CENP-A or CENH3. CENH3 is found in the centromeric nucleosomes with the other histones instead of regular histone H3 and is thought to contribute to both centromeric identity and, through kinetochore formation, to spindle fiber attachment to the chromosome [15].

**Figure 1:**
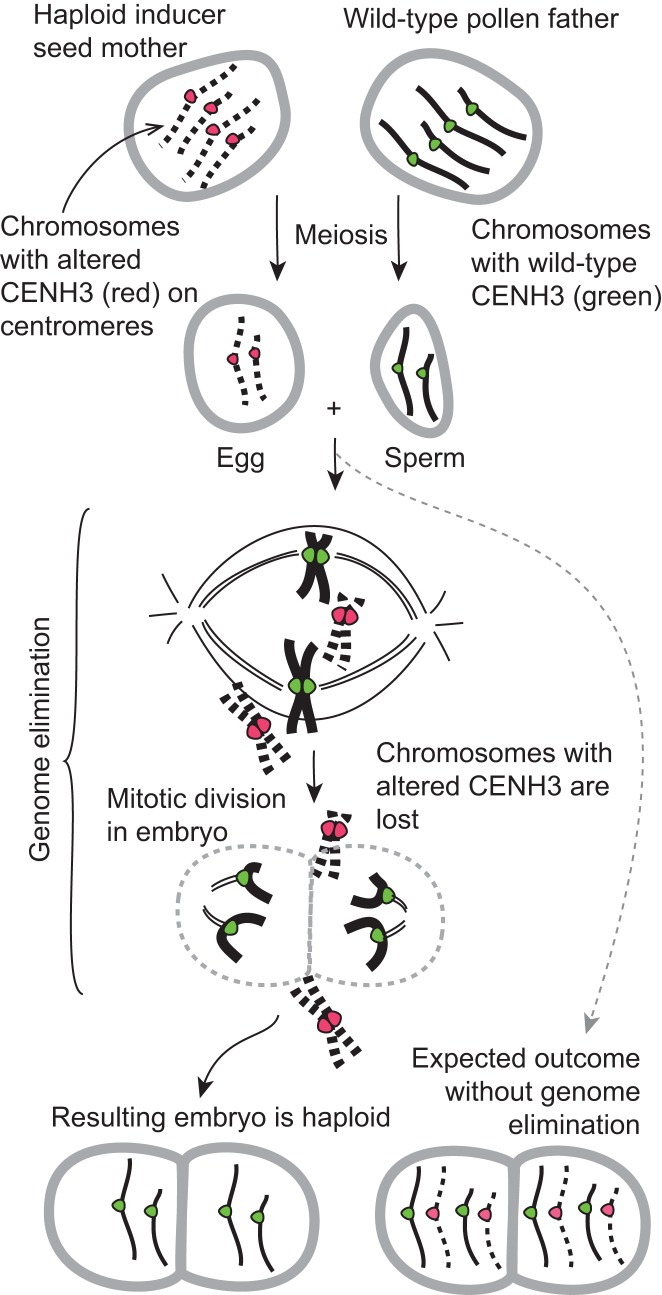
Genome elimination induced by modification of centromeric histone H3(CENH3). An *Arabidopsis* plant becomes a haploid inducer if the native *CENH3* gene is knocked out and complemented with one encoding an altered CENH3. While the chromosomes of the haploid inducer are inherited efficiently upon self-crosses, they are unstable in crosses to a wild-type plant. In the early embryonic mitotic divisions of a hybrid derived from this cross, the chromosomes marked by the defective CENH3 (red) are lost, resulting in a haploid plant of which the nuclear genome derives from the wild-type parent. Diploidization ensues spontaneously or after treatment with spindle inhibitors to produce a fertile dihaploid plant, which is characterized by complete homozygosity. In the lower right, the diploid hybrid produced without genome elimination is depicted. Not shown is the relatively simple step entailing the spontaneous or induced diploidization of the haploid.

With his postdoctoral researcher Ravi Maruthachalam, Simon Chan discovered that a process called genome elimination could be experimentally manipulated in plants [11]. Genome elimination refers to the selective loss of one set of chromosomes from the cell, analogous to the rejection of an organ after transplant. In plants, genome elimination resulting from certain interspecific crosses was described decades ago [16]–[18], but it is limited to occasional, natural occurrences. During genome elimination, the zygote (the product of pollen sperm and egg fusion), inherits both parental chromosome sets but one of the two parental genomes is lost upon the following mitotic divisions. The breakthrough in the Chan lab was the discovery that the experimental alteration of CENH3, by swapping its amino-terminal region and fusing it to green fluorescent protein (GFP) to produce “Tailswap CENH3,” can lead to genome elimination [11]. Chan and colleagues found that genome elimination only occurred when a plant strain with the altered CENH3, referred to as the “Tailswap” haploid inducer, was crossed to a wild-type plant, leading to the elimination of all the Tailswap chromosomes (see Figure 1). Interestingly, the Tailswap genome was stable upon selfing (i.e., when the Tailswap plant fertilized itself), indicating that competition between Tailswap and wild-type centromeres in the hybrid embryo resulted in defective spindle attachment to Tailswap centromeres. Thus, the defective CENH3 mark induces a “weak” state in the genome that results in the failure of Tailswap chromosome segregation when in the presence of a wild-type plant genome. Because there is no change in DNA sequence, this phenotype must result from an epigenetic effect. To date, this event has only been reported in *Arabidopsis*, but given the conserved nature of the perturbed mechanism it is likely to also apply to crop plants.

## Potential Tool for Plant Breeding

Simon Chan and Ravi Maruthachalam realized that genome elimination could prove to be a highly useful tool for plant breeders as it would enable haploid production through a standardized approach. Currently, haploid induction by previously established methods, while desirable, is greatly constrained by genotype and only applicable in selected varieties of certain species [19]. Breeders use hybridization of different accessions and sexual recombination to combine valuable traits. Why would the easy creation of haploid plants by genome elimination be useful? Because the chromosomes of a haploid plant can be doubled by treating it with a spindle apparatus inhibitor or this doubling can occur spontaneously to make a “dihaploid,” which is endowed with complete homozygosity (where the two alleles of each gene are identical). The ability to gain homozygosity in one generation translates into much easier plant breeding [13],[19],[20]. Consider the consequences of self-pollinating an inbred pea line, such as that used by Mendel, versus self-pollinating the highly heterozygous grape strain, Pinot Noir. In the first example, there are no different alleles to assort into new combinations, and the progeny share virtually identical genotypes and phenotypes. In the second, many gene loci carry different alleles, and meiosis reshuffles the allele combinations to result in a variety of phenotypes and genotypes. Efficient agriculture, as well as the analysis of phenotypes, is greatly favored by uniformity, and so most of the crops we propagate by seed are inbred. The only way to propagate the exceptional quality of a heterozygous plant, such as the Pinot Noir grape, is to clone it, that is, to propagate it through cuttings. Indeed, the self-pollination of Pinot Noir takes the ideal combination of alleles in the parent and reshuffles it, resulting in a range of preponderantly agriculturally inferior progeny types.

Many of our annual crops such as pea, soybean, wheat, rice, and peanut are inbred and can be easily propagated via sexual fertilization and seed. Conversely, crops propagated by cuttings or by tubers, such as grapes, banana, fruit trees, potato, and cassava, are highly heterozygous. It is better to reproduce these crops clonally because heterozygosity endows them with hybrid vigor, while inbreeding, in addition to being slow or laborious, is often associated with inferior yield and adaptability.

Based on the known advantages of haploids [19],[20], Chan saw with clarity the gains that could be made by using his genome elimination system as a plant breeding tool. Together with plant breeders and scientists from Africa and South America, Chan and his collaborators conceived a plan to apply genome elimination to cassava and banana. The plan was funded by a joint program between the Bill and Melinda Gates Foundation and the National Science Foundation Plant Genome Program, Basic Research to Enable Agricultural Development (BREAD). This team of researchers proposed that inbred (i.e., dihaploid) cassava or banana is desirable. The reasons for this are well exemplified by the breeding strategy employed for maize. Breeders have developed maize inbred lines that can be cultivated, but are mediocre performers. However, when these lines are hybridized in a favorable combination, the F1 progeny display hybrid vigor and greatly improved adaptability, stress tolerance and increased yield. Thus the inbred lines' desirable traits are combined through hybridization to produce uniform, stress tolerant crops with high yield characteristics [21]. Another advantage of inbreds is easy gene-trait association, because inbreeding enables the genotype to be altered at a specific locus, while keeping the rest of the genome uniform [22]–[24]. Once a gene is connected to a desirable trait, breeding can rely on the use of molecular markers, enabling selection at the seedling stage and in the absence of environmental conditions that may be expensive or difficult to establish.

Currently, the breeding of new banana and cassava varieties is largely empirical and entails crossing two highly heterozygous individuals and selecting desirable types in their progeny to cultivate through clonal propagation. Little is known about which loci determine key agronomic and quality traits. Due to their long reproductive cycle, the inbreeding of these crops is difficult and lengthy at best, most often impossible. Facile haploid induction would also enable plant researchers to exploit the recent genome sequences for these staple food species [25],[26] to connect gene to trait, facilitating the use of molecular markers and thus rapid and efficient breeding cycles. Additionally, new varieties could be produced through the hybridization of selected inbreds: since all resulting F1s are identical these varieties could also be stored and distributed as seed, overcoming storage and distribution problems currently affecting banana and cassava.

## Simon Chan's Legacy

Unfortunately, in the middle of this project, on August 22, 2012, Simon Chan died from complications caused by a longstanding illness. Upon his return from an enthusiastic trip to visit collaborators in Africa and to explore the possible applications of genome elimination, his health rapidly deteriorated. Throughout his illness, Simon remained optimistic about the possible applications of his work to the breeding and propagation of staple food crops. Looking beyond the development of dihaploid strains, Simon envisaged genome elimination as a multipurpose genetic “powertool” that could facilitate basic plant biology research, as well as help to engineer applied crop traits, particularly for the benefit of developing world agriculture and of food security in Africa. The potential of this technology has been demonstrated by the production of clonal seeds (seeds that reproduce the genotype of the hybrid parent) [12]; the rapid construction of mapping resources (recombinant inbred lines) [14]; and the rapid assembly of chromosome substitution lines (molecular breeding) [13]. While these potential applications have been initially demonstrated in the model plant *Arabidopsis*, there is confidence that their translation to economic crops should be forthcoming.

The collaborating project on cassava and banana continues under the direction of Anne Britt (University of California at Davis). The application of this technology beyond *Arabidopsis* will need CENH3 to be manipulated in the target crop. Because the altered CENH3 acts in a recessive fashion [11], engineering a haploid inducer requires knocking out the endogenous *CENH3* gene (or, rarely, *CENH3* genes), and then complementing the endogenous gene with one encoding an altered CENH3. The small size of the *CENH3* gene makes it hard to find variants in mutagenized populations. Silencing *CENH3* by RNAi or altering its function through mutation or through the expression of dominant-negative forms of the gene may be effective, but have not yet been reported. However, recent progress with targeted nucleases [27],[28] may facilitate the task of producing haploid inducers in cassava, banana, and other crops by allowing the targeted manipulation of the *CENH3* gene.

The rewards connected to implementing this technology makes overcoming these hurdles a relatively small price to pay. The community of tropical crop scientists looks forward to success in this project, not least because it would allow Simon Chan's example of successfully addressing a challenging problem in basic science, then leveraging it into practical methods likely to benefit the large fraction of humanity that remains unprivileged, to become his legacy.

## Abbreviations

GM: genetically modified
